# Design, Analysis, and Optimization of a Plasmonic Slot Waveguide for Mid-Infrared Gas Sensing

**DOI:** 10.3390/nano12101732

**Published:** 2022-05-18

**Authors:** Parviz Saeidi, Bernhard Jakoby, Gerald Pühringer, Andreas Tortschanoff, Gerald Stocker, Jasmin Spettel, Florian Dubois, Thomas Grille, Reyhaneh Jannesari

**Affiliations:** 1 Institute for Microelectronics and Microsensors, Johannes Kepler University, 4040 Linz, Austria; bernhard.jakoby@jku.at (B.J.); gerald.puehringer@jku.at (G.P.); reyhaneh.jannesari@jku.at (R.J.); 2 Silicon Austria Labs GmbH, Europastr. 12, 9524 Villach, Austria; andreas.tortschanoff@silicon-austria.com (A.T.); jasmin.spettel@silicon-austria.com (J.S.); florian.dubois@silicon-austria.com (F.D.); 3 Infineon Technologies Austria AG, Siemensstr. 2, 9520 Villach, Austria; gerald.stocker@infineon.com (G.S.); thomas.grille@infineon.com (T.G.)

**Keywords:** plasmonics, slot waveguide, sensing applications, optical simulation, mid-infrared region

## Abstract

In this work, we investigated the optimization of a plasmonic slot waveguide (PSWG) in the mid-IR region particularly for a representative wavelength of 4.26 µm, which is the absorption line of CO_2_ and thus particularly relevant for applications. We analysed the mode features associated with metal-dielectric-metal (MDM), dielectric-metal-dielectric (DMD), and truncated metal film (TMF) structures with respect to the considered PSWG. Subsequently, the mode features of the PSWG were considered based on what we outlined for MDM, DMD, and TMF structures. Furthermore, as confinement factor and propagation length are two crucial parameters for absorption sensing applications, we optimized the PSWG based on a figure of merit (FOM) defined as the product of the aforementioned quantities. To characterize the propagation length, the imaginary part of the effective mode index of a guided mode was considered, leading to a dimensionless FOM. Finally, we investigated the PSWG also for other wavelengths and identified particularly attractive wavelengths and geometries maximizing the FOM.

## 1. Introduction

The mid-infrared (mid-IR) region has attracted great attention owing to a wide range of applications including thermal imaging and infrared spectroscopy [1,2] as it contains the absorption lines of several gases such as CO_2_, CO, and CH_4_ [3,4,5]. A waveguiding mechanism, which is particularly applicable in the mid-IR region, is plasmonics, which represents an important subfield of photonics dealing with the excitation, manipulation, and utilization of surface plasmon polaritons (SPPs) [6]. Plasmonics can be used to enhance the mid-IR sources, sensors, and detectors for applications such as chemical sensing [7]. The coupling between surface localized light waves along with a metal-dielectric interface and free-electron oscillations in the metal creates SPPs, which are a particular class of guided electromagnetic waves [8,9]. The excitation of SPPs allows for efficient light-matter interaction [10,11], resulting in an excellent performance of plasmonic devices in many applications including optical detectors [12,13], optical modulators [14,15,16], filters [17], and sensors [18,19]. The working principle of SPP-based sensors is based on the detection of absorption at the interface between metal and dielectric (e.g., a gas sample containing an analyte which interacts with an evanescent field created on the metal surface) [20]. SPPs have been successfully utilized in sensors for a variety of applications ranging from medical and biochemical applications to environmental monitoring. Among plasmonic devices, plasmonic waveguides are particularly promising candidates for future nanophotonic sensing applications because they can provide high field intensity at a metal/dielectric interface and are also suitable to achieve confinement of the modes on the nanometer scale [21]. In general, plasmonic waveguides can be divided into three different types: dielectric-metal-dielectric (DMD), dielectric-metal (DM), and metal-dielectric-metal (MDM) [22] waveguides. The MDM waveguides, which keep the SPP modes confined in the nanometer range, are interesting candidates for a wide range of applications including waveguide couplers [23,24] and power splitters [25]. One of the MDM waveguides is the slot waveguide, which is composed of a small gap region constituting a low refractive index medium, which is surrounded by two metal rails. The working principle of a plasmonic slot waveguide (PSWG) is related to the coupling of two confined modes at the metallic edges of the slot. Although the PSWG and its mode characteristics have been considered in the past, especially in the near-infrared range [26,27,28], there is no report, to the best of our knowledge, on systematic analysis and optimization of PSWG in the mid-IR region. As any asymmetric PSWG features a cut-off slot width and height, it is essential to design them accurately by calculating the optimized geometries of the structure. In this paper, we focus on the optimization of PSWG in the mid-IR region particularly for a wavelength of 4.26 µm, which is the absorption line of CO_2_ and thus associated with a typical application of IR absorption sensors. Below we illustrate a method for accurate optimization of a PSWG that can generally be used in sensors, particularly absorption sensors. The method is based on the mode features of DMD, MDM, and truncated metal film (TMF) structures which are related to the proposed PSWG. In addition, the proposed PSWG is optimized based on the ratio of slot width to the slot height where the slot area is kept constant.

**Figure 1:**
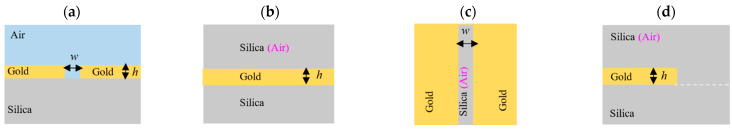
Cross-section view of (**a**) a gold-based plasmonic slot waveguide (PSWG), (**b**) a dielectric-metal-dielectric (DMD) structure, (**c**) a metal-dielectric-metal (MDM) structure, (**d**) a truncated metal film (TMF) structure. In the plasmonic slot waveguide, DMD, and TMF structures, when the gold layer is surrounded by silica, structures are referred to as symmetric structures. In contrast, when air is the upper cladding and silica is the substrate, structures are referred to as asymmetric structures. The white dashed line in (**d**) indicates the interface between air and silica regions when we consider an asymmetric structure.

The proposed PSWG as indicated in Figure 1a, consists of a gold layer located on a silica substrate, and the upper cladding is assumed to be air. In the sensing application, this region will be filled with the analyte containing, e.g., CO_2_. To form the waveguide, a small slot is etched downward into the gold layer. As shown in Figure 1b, the DMD plasmonic structure consists of two half-spaces of dielectric media (silica and air) which are separated by a thin gold region. In this structure, the gold layer has the same thickness as the gold layer in the PSWG. In contrast to the DMD plasmonic structure, the MDM plasmonic structure (shown in Figure 1c) is composed of two infinitely thick gold regions, separated by a thin dielectric medium (silica or air). The corresponding MDM plasmonic structure has the same dielectric thickness (*w*) as the slot width of the PSWG (Figure 1a). Furthermore, as shown in Figure 1d, the gold thickness in the TMF structure corresponds to the gold height of the PSWG. In the following sections, we numerically analyse the mode characteristics of DMD, MDM, and TMF structures associated with the proposed PSWG. The analysis of the associated structures, in particular, allows to identify the border between the guided and leaky modes. We note that in the PSWG, DMD, and TMF structures, when the gold layer is entirely embedded in the silica region, the structures are symmetric, while for air as upper cladding, the structures are considered as an asymmetric structure.

In addition, we optimize the proposed PSWG using a figure of merit (FOM) which is defined based on confinement factor and propagation length, which are two important parameters for absorption sensing applications.

## 2. Methodology

In order to calculate the eigenmode of the plasmonic structures at 4.26 µm, the mode solvers were implemented in COMSOL Multiphysics. To ensure negligible contributions from spurious back reflection, the computational domain was selected as sufficiently large. Furthermore, to absorb outgoing waves and prevent spurious reflections, scattering boundary conditions were applied to all outer boundaries of the simulation domain. Boundary mode analysis using ports at the right and left sides of the computational domain was used for our MDM and DMD structures, while for the truncated metal film and PSWG structures, conventional modal analysis was applied. The real and imaginary parts of the complex permittivities for gold and silicon oxide are $\varepsilon_{gold}=-848.18+62.5i$ and $\varepsilon_{silica}=1.91+0.0012i$ which were taken from [29] and [30], respectively. To consider the effect of gas molecules, the imaginary part of the refractive index of the sensing medium’s material (e.g., CO_2_ in our case) would have to be added to the simulation model. However, the FOM is an intrinsic property of the waveguide and by definition does not include attenuation effects due to the presence of, e.g., CO_2_ in the sensing medium and therefore CO_2_-specific absorption is not part of this particular calculation. Note that the sensitivity to CO_2_ is indirectly considered in the FOM in terms of the confinement factor, though. In the simulation model, the domains associated with silica and air (except for the slot area) were meshed utilizing a triangular mesh, whereas the gold rails and slot region were meshed using mapped elements. In order to achieve precise results, the grid sizes in the computational domains were selected to be as fine as possible based on our computational resources.

## 3. Absorption and Figure of Merit

The sensing mechanism of waveguides used as absorption sensors can be described in terms of the Beer-Lambert law [31]

(1) $$I=I_{0}e^{-\alpha c\Gamma L},\;$$

where I  and $I_{0}$ denote the measured intensity and the initial intensity at a particular CO_2_ concentration c, respectively. α and Γ represent the specific absorption coefficient and confinement factor, respectively. L is the interaction path. Based on Equation (1), it can be seen that the confinement factor Γ plays a major role in the sensitivity of the sensor aiming at determining the concentration c. However, a spurious effect that is not directly included in Equation (1) is the intrinsic attenuation of the guided wave which is not associated with the targeted absorption in the analyte but still leads to an unwanted decrease of the measured intensity. This attenuation is mainly due to the absorption of electromagnetic energy in the gold regions. Therefore, it is desirable to keep this intrinsic attenuation as small as possible. The intrinsic attenuation can be described in terms of the so-called propagation length *L_SPP_* denoting the length, after which the intensity of the guided mode decreases by a factor of 1/e. The confinement factor (Γ) and *L_SPP_* have been discussed in detail in our earlier paper [32].

Thus, a sensor with high Γ and high *L_SPP_* is desired resulting in higher sensitivity of the sensor. It turns out that designing a waveguide frequently represents a trade-off between $L_{SPP}$ and Γ for plasmonic waveguides and in order to optimize the behavior we define a dimensionless Figure of Merit (*FOM*) as:

(2) $$FOM=\frac{\Gamma }{N_{eff}^{imag}}$$

where the $N_{eff}^{imag}$ is the imaginary part of the effective mode index of the guided mode which is linked to the propagation length. In particular, smaller intrinsic attenuation corresponds to increased *L_SPP_* and reduced $N_{eff}^{imag}$. In the last section, we report on the optimization of the proposed PSWG with respect to this *FOM*.

## 4. Results and Discussion

### 4.1. Analysis of DMD Plasmonic Structure

**Figure 2:**
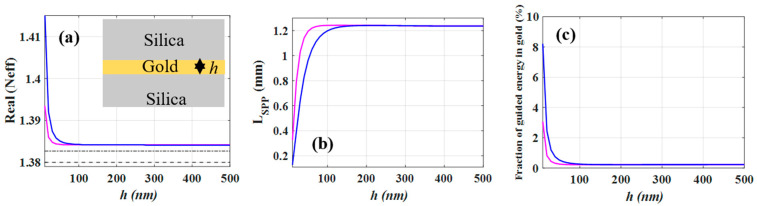

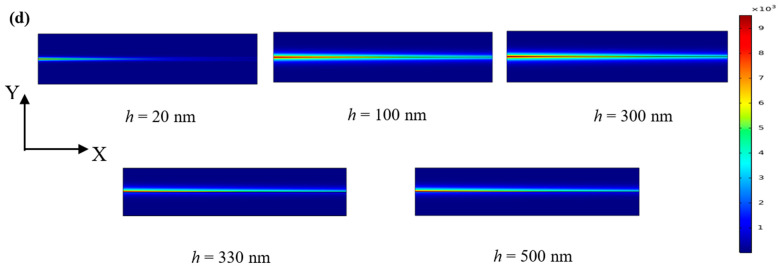
(**a**) The real parts of effective mode index ($N_{eff}$) of symmetric silica-gold-silica (blue curve) and asymmetric air-gold-silica (magenta curve) DMD structures as a function of gold thickness (*h*). The dashed line and the dash-dotted line show the refractive index of silica and the effective mode index of a single silica-gold interface. (**b**) Propagation length (*L_SPP_*) of DMD structures versus gold thickness. (**c**) The fraction of guided electromagnetic energy in gold versus *h*. (**d**) Illustration of the electric field distribution (norm E) for different gold thicknesses for silica-gold-silica (symmetric structure). The lateral and vertical simulation domain sizes in (**d**) are 1 mm and 200 µm, respectively. Blue regions correspond to low field intensities and red regions to larger intensities.

The real parts of the effective mode index ($N_{eff}$) of the guided mode (TM polarization) for both silica-gold-silica (symmetric) and air-gold-silica (asymmetric) DMD structures as a function of gold thickness, *h*, are plotted in Figure 2a. It can be observed that for both structures, as *h* decreases, $N_{eff}$ increases. The $N_{eff}$ for the asymmetric DMD structure (magenta curve) shows a lower value than that of the symmetric one (blue curve) for *h* smaller than 70 nm while for higher values they virtually have the same values. Moreover, for both structures, increasing *h* leads to vanishing coupling of the SPP mode between two gold-silica (air) interfaces such that their $N_{eff}$ asymptotically approaches to that of an SPP mode associated with a single gold-silica interface (dash-dotted line in Figure 2a).

Figure 2b indicates the *L_SPP_* of both DMD structures as a function of *h*. For both DMD structures, the *L_SPP_* increases rapidly with the increase of h and then saturates. Figure 2c clearly shows that increasing h leads to a lower fraction of the guided electromagnetic energy in the gold region. The electric field distribution of the symmetric DMD structure for different gold thicknesses has been illustrated in Figure 2d. For instance, for *h* = 20 nm, due to strong coupling between two gold-silica interfaces, the amount of guided field intensity in the gold layer as plotted in Figure 2c, is higher than for other thicknesses resulting in lower *L_SPP_* (see Figure 2b). We note that for *h* < 20 nm, the coupling between two gold-silica interfaces is even stronger. Increasing *h* up to 150 nm will lead to less coupling between the two interfaces, thus increasing the *L_SPP_*. Nevertheless, a further increasing of *h* has no impact on the energy transmitted in gold layers thus leading to a virtually constant *L_SPP_*.

### 4.2. Analysis of MDM Plasmonic Structure

**Figure 3:**
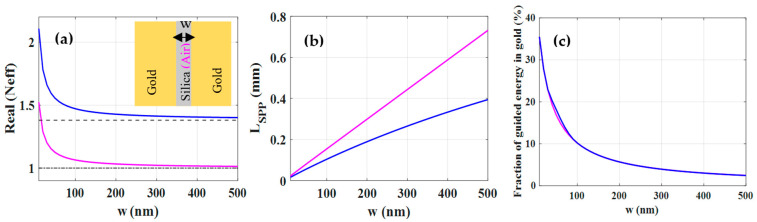
(**a**) The real parts of effective mode index ($N_{eff}$) of gold-silica-gold (blue curve) and gold-air-gold (magenta curve) MDM structures as a function of silica (air) thickness (*w*). The dashed line and dash-dotted line show the refractive index of silica and air, respectively. (**b**) The propagation length of MDM structures versus *w*. (**c**) The fraction of guided electromagnetic energy in gold as a function of *w*.

The $N_{eff}$ of the guided mode of both, gold-silica-gold and gold-air-gold MDM structures has been indicated in Figure 3a. Like for the DMD structures, the $N_{eff}$ of MDM structures have a higher value as *w* decreases. In addition, $N_{eff}$ asymptotically approaches the refractive index of the dielectric region (silica or air) as *w* increases indicating the concentration of the field energy in the dielectric region. As shown in Figure 3b, the *L_SPP_* of both MDM structures increases with the increase of *w* because the amount of guided electromagnetic energy inside the gold decreases as depicted in Figure 3c, leading to a decrease of ohmic losses. Although Figure 3c indicates similar behavior for the guided energy in gold for both structures, the *L_SPP_* of the gold-silica-gold MDM structure for a given *w* is lower than that of the gold-air-gold one which is attributed to the absorption loss of silica in particular. A comparison between the *L_SPP_* of MDM and DMD structures for a given thickness of the central region shows a smaller *L_SPP_* for the MDM structures. This is due to the fact that the coupling of the single-interface SPPs in the MDM structure is stronger than that of the DMD structure because in the former the coupling is created via a dielectric while in the latter, through the metal [28]. In other words, the guided transmitted energy inside gold in MDM structures is higher (see Figure 3c) than that of otherwise comparable DMD structures (see Figure 2c), resulting in a lower *L_SPP_* for MDM structures compared to the DMD structures.

### 4.3. Analysis of Plasmonic Truncated Metal Film (TMF) Structure

**Figure 4:**
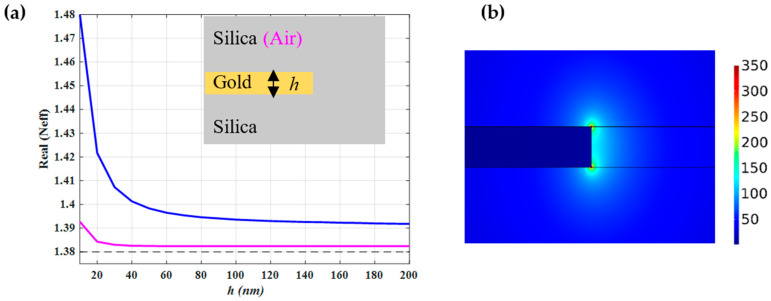
(**a**) The real parts of the effective mode index ($N_{eff}$) of the fundamental edge mode of a truncated gold layer when it is embedded as whole in the silica region (blue curve) and when the upper and lower cladding are air and silica, respectively (magenta curve). The dashed line represents the refractive index of the substrate. (**b**) The mode profile of the edge mode which is supported by a truncated gold embedded in silica where the gold thickness is 10 nm. The right-side rectangle in the picture is created to refine the mesh around the edge of the gold-silica interface.

The $N_{eff}$ for a truncated gold layer which is fully embedded in a silica region (symmetric structure) and for the case, where it is placed in the interface between air and silica (asymmetric structure), is shown in Figure 4a. Both curves approach an asymptotically constant value with increasing *h*, although the symmetric structure shows a higher value of $N_{eff}$ compared to the asymmetric structure for a given *h*. Figure 4b illustrates the fundamental edge mode of a truncated gold film layer when the gold film is embedded in a silica region. As can be observed, the SPP mode is confined at the edge of the gold film at its interface with silica. In addition, due to the singular behavior of the electric field near the sharp edges [33], the maximum intensity of the SPP mode occurs at the two corners of the edge of the gold film.

### 4.4. Analysis of Symmetric and Asymmetric Plasmonic Slot Waveguides

**Figure 5:**
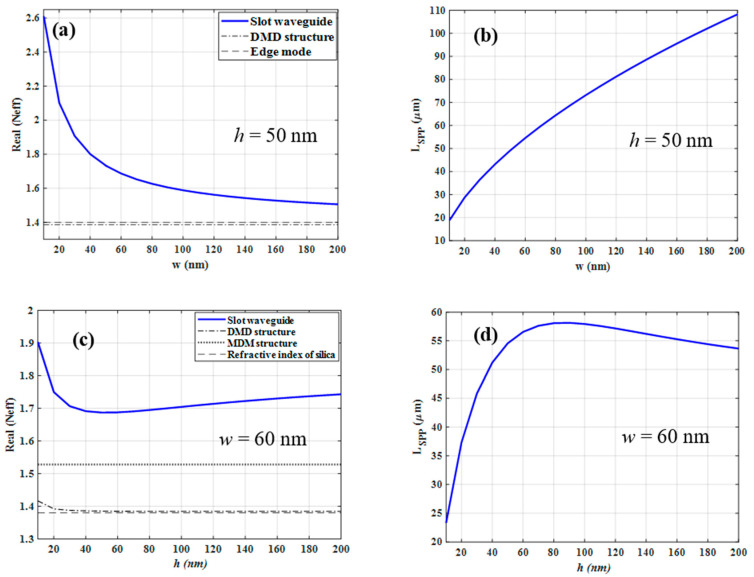
(**a**) The variation of effective mode index ($N_{eff}$) of the fundamental mode of a symmetric PSWG versus the slot width (*w*) when the gold thickness is fixed to 50 nm. (**b**) The propagation length of the symmetric PSWG as a function of the slot width *w*. (**c**) The effective mode index of the fundamental mode of the symmetric PSWG as a function of the gold thickness (*h*) when the gap width is fixed to 60 nm. (**d**) The propagation length of the symmetric PSWG as a function of the gold thickness *h*.

Here we investigate the mode characteristics of symmetric and asymmetric gold-based PSWGs. As an example, the following dimensions are used for the reference structure, *w* = 60 nm and *h* = 50 nm. First, we investigate the symmetric one where the gold-based slot waveguide is entirely embedded in silica. As indicated in Figure 5a, the real part of $N_{eff}$ shows a similar asymptotic behavior for increasing w as the above-mentioned structures, where *h* is fixed to 50 nm. As *w* decreases, the $N_{eff}$ increases which is due to the fact that the fraction of modal intensity in the gold layers increases, thus also decreasing *L_SPP_* (see Figure 5b). As *w* increases, the $N_{eff}$ approaches to the effective mode index of the edge mode which is a bit higher than that associated with the silica-gold-silica DMD structure. In particular, for increasing *w* the mode becomes less confined into the slot region which originates from the reduced coupling between the two edge modes confined at the two gold layers.

The variations of $N_{eff}$ and *L_SPP_* of symmetric PSWG as a function of *h,* where *w* is fixed to 60 nm, are shown in Figure 5c,d, respectively. As *h* decreases, $N_{eff}$ increases and *L_SPP_* decreases which is due to the fringing fields which are confined at the four corners of the gold-silica interfaces [28]. The changes of $N_{eff}$ for symmetric plasmonic gold-based slot waveguides versus *w* and *h* show that it always features higher $N_{eff}$ values than the corresponding MDM, DMD, and truncated gold film structures and never crosses them. Therefore, the symmetric PSWG always supports a guided mode and has no cut-off.

**Figure 6:**
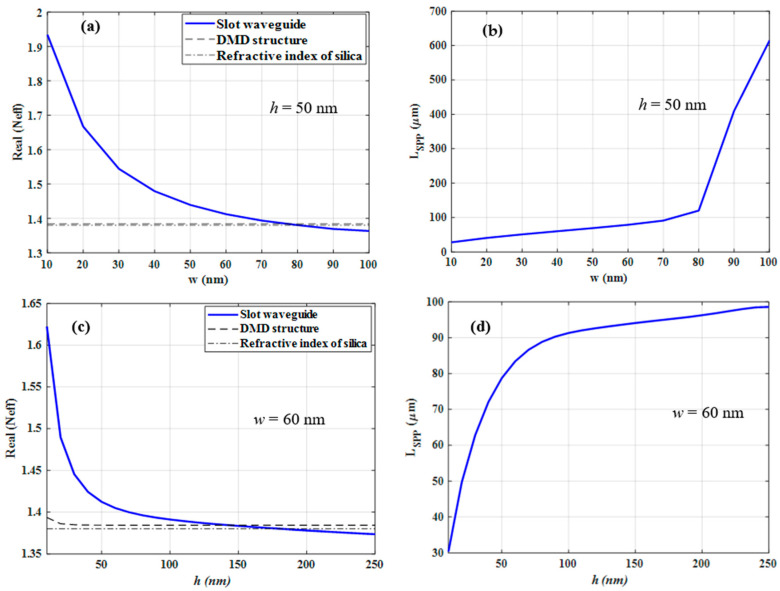
(**a**) The variation of the effective mode index ($N_{eff}$) of the fundamental mode of the asymmetric PSWG as a function of the slot width (*w*) when the gold thickness is fixed to 50 nm. (**b**) The propagation length of the asymmetric PSWG as a function of the slot width *w*. (**c**) The effective mode index of the fundamental mode of the asymmetric PSWG as a function of the gold thickness (*h*) when the gap width is 60 nm. (**d**) The propagation length of asymmetric PSWG as a function of the gold thickness *h*.

For the asymmetric PSWG, the gold-based slot waveguide is located on top of the silica substrate and its upper cladding is air. As can be seen from Figure 6a, as *w* increases, the $N_{eff}$ decreases as in the symmetric case. However, in contrast to the symmetric case, the SPP mode in the asymmetric PSWG is not always bound and will be leaky if the slot width exceeds a certain value (the so-called cut-off slot width). Normally, the cut-off slot width of any asymmetric PSWG can be determined based on the $N_{eff}$ of the corresponding edge mode [28]. In our case the $N_{eff}$ of the edge mode is close to the refractive index of the silica substrate and is smaller than that of the asymmetric silica-gold-air DMD structure. Therefore, there is a cut-off slot width *w_c_* and for *w* > *w_c_* the mode begins to leak into spurious guided modes of the silica-gold-air DMD structure which are not confined to the slot area. Moreover, further increase in *w* leads to an approach to the refractive index of the silica substrate which represents another independent cut-off limit *w_s_*. For *w* > *w_s_*, the modal intensity pattern changes so that more field energy is contained in the silica substrate corresponding to leakage into the substrate. The *L_SPP_* of the fundamental mode of the asymmetric PSWG versus *w* is depicted in Figure 6b. Increasing *w* will lead to an increase of *L_SPP_* because as *w* increases the confinement of the SPP mode into the gap region becomes less. Therefore, the more field penetrates in the substrate, the less field is confined in the gap region resulting also in less electromagnetic energy in the gold. Interestingly, the *L_SPP_* even increases when the mode is leaky which is due to the fact that the dominant loss mechanism in the structure is the material loss in gold [32]. The comparison between the *L_SPP_* of the asymmetric PSWG (see Figure 6b) and the symmetric one (see Figure 5b) for the same *w* shows that the *L_SPP_* of the asymmetric one increases faster than that of the symmetric one.

Figure 6c shows the variation of $N_{eff}$ as a function of *h* for fixed width *w* = 60 nm. The $N_{eff}$ asymptotically decreases as *h* increases. Normally, as *h* increases, the $N_{eff}$ of the fundamental mode of the asymmetric PSWG approaches that of the MDM structure where the dielectric region corresponds to the slot region. However, in our case, the $N_{eff}$ of the fundamental mode which is supported by the gold-air-gold MDM structure (see Figure 3a) is smaller than that of the silica-gold-air DMD structure. Thus, there is a cut-off metal thickness *h_c_* and for *h > h_c_* the mode cannot be considered as a guided mode. Furthermore, the $N_{eff}$ of the fundamental mode of the asymmetric PSWG is also smaller than the refractive index of the substrate. Therefore, there is a cut-off metal thickness which is due to the substrate, *h_s_*, and for *h* > *h_s_* the modal field pattern changes and the mode increasingly leaks into the silica substrate. The *L_SPP_* of the fundamental mode of the asymmetric PSWG as a function of *h* is plotted in Figure 6d. Increasing *h* will lead to an increase of *L_SPP_* even when the mode becomes leaky due to the same reason mentioned above when considering the increase of *w*.

### 4.5. Optimization of the Proposed PSWG

**Figure 7:**
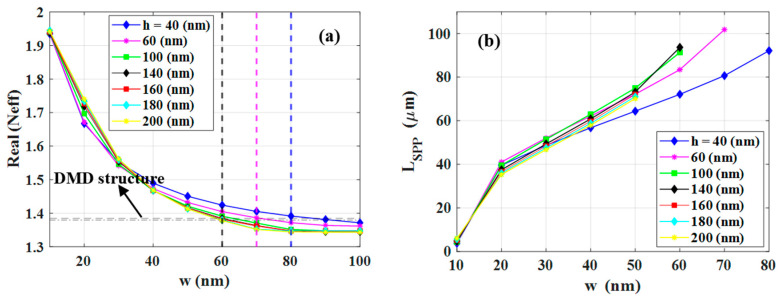
(**a**) The real part of the effective mode index ($N_{eff}$) of the plasmonic slot waveguide (PSWG) as a function of the slot width w for different slot heights *h*. The horizontal dashed line shows the effective mode index of the silica-gold-air DMD structure, while the dash-dotted line below indicates the refractive index of the silica substrate. Three vertical black, magenta and blue dashed lines represent the cut-off slot widths for h = 40, 60, as well as 100 and 140 (featuring the same cut-off). (**b**) The propagation length of the fundamental guided mode of the PSWG as a function of the slot width.

In this section, we present the optimization of the PSWG (see Figure 1a) based on what we outlined in the previous sections. Although our optimization is performed for a wavelength of 4.26 µm, which is the absorption band of CO_2_, it also works similarly for the other wavelengths to obtain precise geometries of PSWGs. The optimization is based on the FOM defined in Section 3. The $N_{eff}$ of the fundamental SPP mode of the PSWG as a function of *w* for different *h* has been depicted in Figure 7a. Based on the results discussed above, the horizontal dashed line, which shows the $N_{eff}$ of the corresponding silica-gold-air DMD structure, is the limit for the guided mode. Below this line, the mode field patterns change significantly representing leaky modes. Moreover, the three vertical dashed lines with different colors indicate the cut-off slot widths for different *h*. The blue and magenta dashed lines indicate the cut-off slot widths for *h* = 40 and 60 nm, which are 80 and 70 nm, respectively. Furthermore, the black line shows the cut-off slot width for *h* = 100 and 140 nm, which is 60 nm in both cases. Therefore, we only consider modes that are above the horizontal dashed line representing guided modes. The *L_SPP_* of the guided mode as a function of *w* for different *h* is shown in Figure 7b. As already demonstrated in Figure 6b, the *L_SPP_* increases with the increase of *w* which is the result of a reduced energy fraction in the gold layers. Due to the maximum amount of electromagnetic energy in gold layers, the lowest *L_SPP_* occurred for *w* = 10 nm. For this particular *w*, increasing *h* has no significant effect on *L_SPP_*.

**Figure 8:**
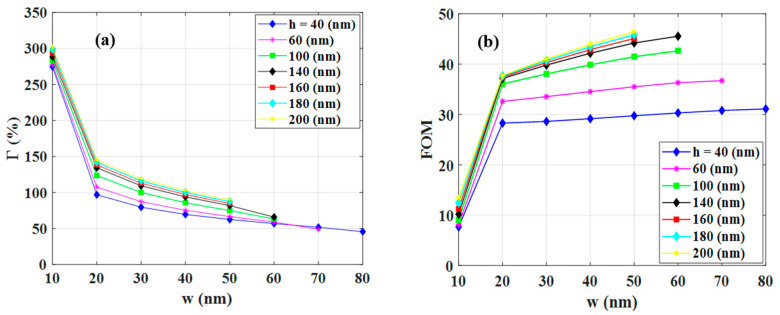
(**a**) The confinement factor Γ and (**b**) the FOM for the plasmonic slot waveguide as a function of slot width w for different slot heights.

**Figure 9:**
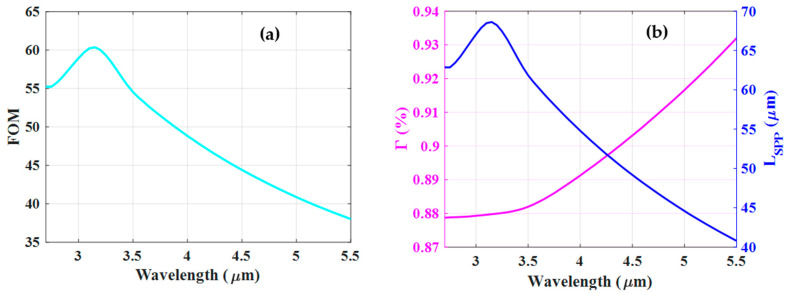
(**a**) The FOM of the optimized plasmonic slot waveguide (w=50 nm, h=200 nm) as a function of the wavelength. (**b**) The corresponding confinement factor and propagation length.

In Figure 8a, the calculated confinement factor Γ of the PSWG as a function of slot width has been plotted. As one can see, Γ shows the highest value for *w* = 10 nm because of the tight confinement of the SPP mode in the gap region. As a consequence, more electromagnetic energy can penetrate into the gold layers resulting in the lowest propagation length (see Figure 7b). However, as the slot width increases the mode becomes less confined in the slot area and more field penetrates the substrate, thus decreasing Γ. The FOM of the PSWG is depicted in Figure 8b. The optimal FOM achieved is 46.5 which is associated with *w* = 50 nm and *h* = 200 nm. The corresponding Γ and *L_SPP_* are approximately 90% and 70 µm, respectively. Moreover, the performance of the optimized PSWG in the wavelength range of 2.7–5.5 µm has been investigated. As it is presented in Figure 9a, the FOM has a maximum value of 60.2 at a wavelength of 3.1 µm. Figure 9b depicts the corresponding Γ and *L_SPP_*. As can be observed clearly, the FOM indicates similar behavior as the *L_SPP_* diagram, which confirms that the material properties have a major effect on the FOM.

In addition to the previous study described in detail above, the optimized ratio of the slot width to slot height (R=w/h) for a constant slot area (A) is investigated for both wavelengths, i.e., 4.26 µm and 3.1 µm. As the DMD cut-off is mainly close to the refractive index of the substrate (see Figure 7a), considering the refractive index of the silica substrate as a cut-off is considered to be a good approximation.

**Figure 10:**
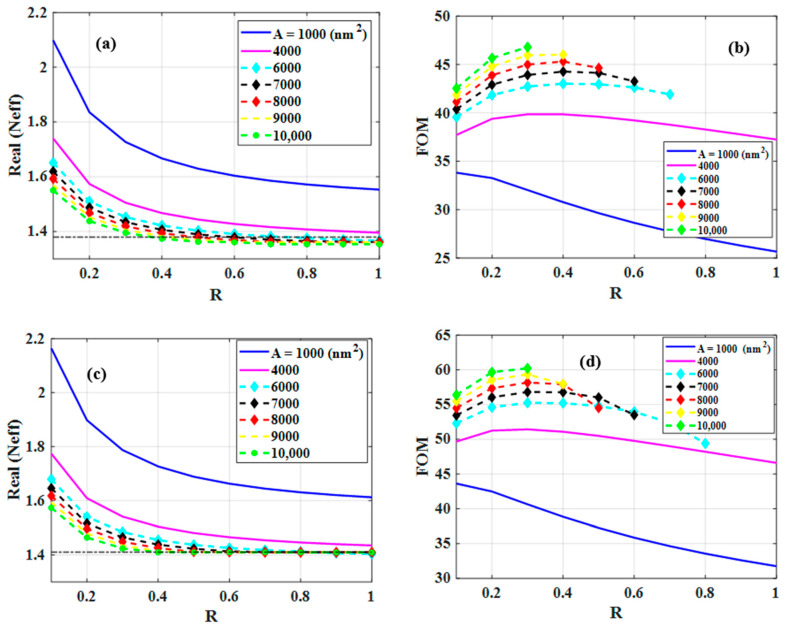
The real part of the effective mode index ($N_{eff}$) of the plasmonic slot waveguide (PSWG) as a function of ratio between slot width and height (R) for different values of slot area (*A*) for a wavelength of (**a**) 4.26 µm and (**c**) 3.1 µm. The horizontal dashed line represents the refractive index of the silica substrate. The FOM of the fundamental guided mode of the PSWG versus R for different A for a wavelength of (**b**) 4.26 µm and (**d**) 3.1 µm.

The variations of $N_{eff}$ versus R for different values of A have been depicted in Figure 10a,c for target wavelengths of 4.26 µm and 3.1 µm, respectively. The horizontal dashed line in both figures shows the refractive index of the substrate indicating a cut-off where below it the mode is not a guided mode anymore due to high field penetration in the silica substrate. In both figures, the two blue and magenta solid lines which belong to A = 1000 ${\mathrm{nm}}^{2}$ and 4000 ${\mathrm{nm}}^{2}$ respectively, stay above the cut-off for all values of *R*, while, for A=6000, 7000, 8000, 9000 and 10,000 ${\mathrm{nm}}^{2}$, the values of R larger than 0.7 (0.8), 0.6, 0.5, 0.4 and, 0.3 for a wavelength of 4.26 µm (3.1 µm) respectively, yield an effective refractive index below the dashed line. Hence, they are not guided modes anymore. Figure 10b,d illustrate the FOM of the guided modes of corresponding structures. Here, the optimal FOM for 4.26 µm is 46.8 which corresponds to R=0.3 and $A=10,000\;nm^{2}$ which is very close to the one we obtained in the first part ($R=0.25,\;A=10,000\;{\mathrm{nm}}^{2})$. The optimal FOM for a wavelength of 3.1 µm is 60.2 (Figure 10d). The optimized parameters (*R* = 0.3 and *A* = 10,000 ${\mathrm{nm}}^{2}$) for the proposed PSWG at both wavelengths are identical. Therefore, the proposed PSWG can be a good candidate for the applications where the target wavelengths are 3.1 µm and 4.26 µm.

As the PSWG proposed in this work has the same structure (but with different plasmonic material) as the one proposed in [34], it is interesting to compare the FOMs of these devices which are proposed for the same purpose (i.e., sensing at 4.26 µm which is the absorption line of CO_2_). The obtained FOM for the proposed PSWG in this work is much higher than the one reported in [34], i.e., (3.2). At the same time, we note that the achieved FOM in this work is lower than the FOMs of (276.4 and 70.1) reported in [32], which is related to different sensor structures, though.

### 4.6. Proposed Fabrication Steps for the PSWG

The present investigation focuses on potentially achievable performance and design aspects. Yet we want to indicate some possibilities regarding the fabrication of the proposed PSWG, which can be done using standard CMOS compatible processes as for example chemical vapor deposition (CVD), lithography (spin coating, exposure, development), dry or wet chemical etching and lift-off process. Starting with polished and cleaned single crystalline silicon substrates, the 2 µm thick SiO_2_ can be deposited using low-pressure chemical vapor deposition (LPCVD). The deposition of Au can either be done by means of evaporation or sputtering process. In order to avoid peeling, typically a thin layer of titanium (Ti, 50 nm) is deposited as adhesion promoter between silicon oxide and gold. Depending on the resolution and size of the individual geometries, the structuring of the gold layer can be done with different processes. Lift-off or wet chemical etching processes allow thereby lower resolution than comparable ion beam etching processes. The described slot geometries would most likely require a combination of electron beam lithography (EBL) and ion beam etching to achieve the required high resolution.

## 5. Conclusions

In this paper, the mechanism of guiding mode in a plasmonic slot waveguide (PSWG) is outlined and its structure is optimized. To illustrate the approach, we considered a wavelength of 4.26 µm, which is an absorption line of CO_2_. We first investigated the mode characteristics of DMD, MDM, and TMF structures. Subsequently, mode features of both, symmetric and asymmetric PSWG were analyzed to show the border between guided and leaky modes. We showed that the variation of the effective mode index of the symmetric PSWG as a function of slot width or slot height is always higher than the corresponding MDM, DMD, and TMF structures and never crosses them. Therefore, the symmetric PSWG always features a guided mode. In contrast, the asymmetric PSWG showed a cut-off slot width and slot height where beyond them the mode will be leaky. In addition, based on the obtained characteristics, we optimized the PSWG geometries using a FOM defined as a product of confinement factor and propagation length, which are two essential quantities for sensing applications. The optimized value for the ratio of the slot width to the slot height for a constant slot area was found. The FOM of the optimized structure for the wavelength range of 2.7–5.5 µm shows a maximum for a wavelength of 3.1 µm which is 60.2. The maximum FOM found for a wavelength of 4.26 µm is 46.8. Further investigation of the FOM as a function of the slot width to slot height ratio and slot area at a wavelength of 3.1 µm revealed that the proposed PSWG has the same optimal geometry (*R* = 0.3 and *A* = 10,000 ${\mathrm{nm}}^{2}$) as the one for a wavelength of 4.26 µm. Therefore, the proposed structure is considered to be a candidate for applications where the target wavelengths are 3.1 µm and 4.26 µm. 

## Data Availability

The data presented in this study are available on request from the corresponding author.
